# Long-term kidney outcomes after living donation in older adults: clinical findings and equation-dependent eGFR estimates

**DOI:** 10.1186/s12877-026-07826-8

**Published:** 2026-07-07

**Authors:** Iris Schröter, Claudia Sommerer

**Affiliations:** Department of Nephrology, University Hospital Heidelberg, Im Neuenheimer Feld 162, Heidelberg, D-69120 Germany

**Keywords:** Living kidney donation, Age, Renal function, eGFR formula, Trajectories

## Abstract

**Background:**

Living kidney donation from older individuals is increasingly considered, yet, the long-term trajectories of kidney function and clinical outcomes remain insufficiently characterised. In particular, the impact of different commonly used estimation equations of the glomerular filtration rate (eGFR) on long-term risk assessment in older donors is unclear.

**Methods:**

We analyzed 131 living kidney donors aged ≥ 60 years at the time of donation with a median follow-up of 12 years. Renal function was assessed using the CKD-EPI, FAS, and BIS1 equations at baseline, one year post-donation, and at long-term follow-up. Outcomes included substantial long-term eGFR decline (> 40%), the annual eGFR change after year 1, perioperative complications, long-term comorbidities, donor mortality, and recipient outcomes.

**Results:**

Renal function followed a consistent biphasic pattern across all equations, with an early decline after donation followed by long-term stability. 37.0% of the donors experienced an eGFR decline > 40%, 13.4% >50%). The proportion classified below clinically relevant eGFR thresholds varied substantially depending on the eGFR equation applied, particularly one year after donation. Chronological age, including ≥ 70 years, was not associated with accelerated long-term decline. Impaired renal adaptation at one year, baseline hypertension, higher body mass index, and female sex were independently associated with steeper long-term eGFR decline. Among donors with preserved long-term eGFR (≥ 45 mL/min/1.73 m²), albuminuria identified a subgroup with less favourable renal trajectories. Relevant perioperative complications were rare (2.3%). No donor required renal replacement therapy during follow-up.

**Conclusions:**

In carefully selected donors aged ≥ 60 years, living kidney donation is associated with stable long-term renal function and a low rate of clinically relevant eGFR decline. Long-term risk is primarily determined by biological and cardiometabolic factors rather than by age itself. The interpretation of post-donation kidney function is influenced by the estimation equation applied, highlighting the importance of longitudinal assessment rather than reliance on single threshold-based classifications.

**Trial registration:**

Eudra-CT 2012-003500-12.

**Supplementary Information:**

The online version contains supplementary material available at 10.1186/s12877-026-07826-8.

## Key Points

• Renal donation is safe in selected donors aged 60 or older with stable long-term renal function and low rate of clinically relevant eGFR decline.

• Long-term risk is driven primarily determined by biological and cardiometabolic factors rather than by age itself.

• The Interpretation of post-donation kidney function is strongly dependent on the estimation equation applied.

• In older living kidney donors, the choice of eGFR equation substantially influences both absolute eGFR estimates and clinical classification, with the greatest discrepancies occurring early after donation and in donors with eGFR values close to clinically relevant thresholds.

• Differences between eGFR equations resulted in substantial variation in clinical classification, particularly early after donation and in donors close to clinically relevant thresholds.

• Long-term GFR decline in older donors is slower than expected from age-related decline in the general population.

**Supplementary Information:**

The online version contains supplementary material available at 10.1186/s12877-026-07826-8.

## Background

Kidney transplantation is the optimal therapy for eligible patients with end-stage renal disease. Notably, the demographic profile of both the general population and patients on renal replacement therapy is changing, with older patients (≥ 60 years) representing the fastest-growing group of patients initiating maintenance dialysis in many countries [1].

Interestingly, transplantation of kidneys from older deceased donors into older patients with end-stage renal disease is widely accepted [2, 3]. However, some transplant centers are reluctant to consider transplantation from older living donors [4], despite long-term observational data demonstrating a steady increase in donor age over the last few decades [5]. Recent analyses from Germany likewise confirm the growing proportion of older living kidney donors [6–8].

Several concerns are raised, including whether the perioperative risk is acceptable for the donor, how long-term outcomes - especially renal function - develop in older donors, and whether kidneys from older donors provide sufficient graft function over an acceptable period [9].

Living donation offers several advantages over transplantation from deceased donors, particularly for older recipients [10–15], and has also been associated with favourable patient-reported outcomes such as quality of life and fatigue [7, 16, 17]. Transplants from living donors, even when originating from older donors, usually show immediate function, allowing early discharge and rapid return to regular daily activities. Furthermore, planned surgery is particularly beneficial for older patients, who more frequently present with comorbidities than younger patients. Moreover, living donation enables preemptive transplantation, thereby avoiding dialysis exposure [13].

Donors aged ≥ 60 years represent a distinct group with age-related changes in renal physiology and reduced nephron reserve, which may influence post-donation renal function over extended periods [18, 19]. Long-term follow-up data in older living kidney donors remain limited, as most previous studies have focused on younger donors or short- to mid-term outcomes. In the general population, age-related decline in eGFR is typically reported at approximately 0.5–1.0 mL/min/1.73 m² per year in older adults [20, 21]. Whether the annual decline in kidney function in older living kidney donors is comparable to this physiological age-related decline remains open. In daily clinical practice, assessment of post-donation kidney function is primarily assessed using estimated glomerular filtration rate (eGFR), which directly informs clinical decision-making.

In clinical practice, post-donation kidney function is primarily assessed using estimated glomerular filtration rate (eGFR), which directly informs clinical decision-making. The Chronic Kidney Disease Epidemiology (CKD-EPI) equation is widely used, whereas age-adapted equations such as the Full Age Spectrum (FAS) and Berlin Initiative Study (BIS1) equations have been developed to improve estimation in older adults [22–24]. However, all of these equations were derived from populations with two functioning kidneys. Consequently, equation-dependent differences may influence eGFR levels and classification, particularly in older donors.

Beyond renal function trajectories, long-term clinical outcomes such as cardiovascular events, mortality, and perioperative complications have not been comprehensively analysed in older donors. Therefore, this study focuses on the longterm outcome of living kidney donors aged ≥ 60 years with particular emphasis on post-donation GFR trajectories and their interpretation across different estimation equations.

## Methods

Patients enrolled in the present analysis were participants of the Heidelberg Kidney Donor Study (HeiKiD), a prospective cohort study established to evaluate long-term outcomes after LKD and to enhance shared decision-making and informed consent. The study was approved by the ethics committee of the University Hospital Heidelberg (S104-2011), and all participants provided written informed consent. Data handling complied with the European General Data Protection Regulation.

Eligible donors were aged ≥ 60 years, had completed the pre-donation evaluation, and had at least 5 years of follow-up after donation. Demographic data were obtained via structured questionnaires. Clinical and laboratory data were collected before donation, one year post-donation, and annually thereafter.

The primary objective of this study was to characterise long-term renal function and eGFR decline in living kidney donors aged ≥ 60 years and to determine how these trajectories differ when kidney function is assessed using the CKD-EPI 2021, FAS, and BIS1 Eqs [22–24].

Secondary objectives were to quantify equation-dependent differences in absolute eGFR levels, clinical classification below commonly used thresholds, and the prevalence of substantial long-term eGFR decline (> 40%) across CKD-EPI, FAS, and BIS1. A decline in eGFR of > 40% from baseline to long-term follow-up was defined as clinically relevant, as this threshold is widely used as a surrogate endpoint for substantial kidney function loss in longitudinal studies and has been associated with increased risk of adverse renal outcomes [25, 26]. Additional objectives included the assessment of early post-donation renal adaptation at one year as a marker of long-term renal vulnerability, the identification of donors with accelerated decline based on trajectory analysis, and the evaluation of clinical factors associated with unfavourable long-term renal outcomes. Finally, perioperative safety, long-term donor comorbidities, donor survival, and recipient outcomes were examined.

### Statistical analysis

Continuous variables are presented as mean ± standard deviation or median with interquartile range (IQR), as appropriate.

Renal function trajectories were analysed longitudinally using absolute annual eGFR slopes between one year after donation and long-term follow-up (mL/min/1.73 m²/year), calculated separately for CKD-EPI, FAS, and BIS1. Group differences in slope distributions were assessed using Wilcoxon rank-sum or Kruskal–Wallis tests, as appropriate. Donor selection at our center was based on comprehensive clinical assessment, while FAS and BIS1 were applied retrospectively for study purposes only and did not influence donor selection.

Equation-dependent classification variability was evaluated using a clinically relevant threshold of eGFR < 45 mL/min/1.73 m². This threshold corresponds to CKD stage 3b as defined in international guidelines [27, 28]. Agreement between equations was quantified by pairwise discordance rates (CKD-EPI vs. FAS, CKD-EPI vs. BIS1, FAS vs. BIS1) and by multi-equation concordance patterns (all ≥ 45, all < 45, CKD-EPI only < 45, mixed). Sensitivity analyses were performed using alternative cut-offs (40, 45, and 50 mL/min/1.73 m²), categorising donors as never below, transiently below, persistently below, or late crossing of the respective threshold between one year and long-term follow-up.

Renal adaptation at one year was defined as the ratio of eGFR at year 1 relative to baseline and donors were stratified into good (≥ 70%), intermediate (60–69%), and poor (< 60%) adaptation groups. Long-term slopes and proportions of substantial eGFR loss were compared across adaptation strata. A decline in eGFR of > 40% was considered clinically relevant, as this threshold is widely used as a surrogate endpoint for substantial kidney function loss in longitudinal nephrology studies [25, 26].

Rapid decline was defined a priori as an annual eGFR decrease > 3 mL/min/1.73 m²/year. Equation-specific prevalence and concordance of rapid decline were quantified. Donors were classified as rapid by all equations, non-rapid by all equations, equation-specific rapid, or mixed.

To evaluate potential over-classification at one year, a clinically oriented “CKD-EPI alarm” was defined as CKD-EPI < 45 mL/min/1.73 m² at one year, and “false alarm” as recovery to ≥ 45 at long-term follow-up. Long-term outcomes (advanced kidney dysfunction, albuminuria, cardiovascular events, death, and composite adverse outcome) were compared between alarm and non-alarm groups.

Determinants of long-term eGFR decline were analysed using multivariable linear regression with annual slope as the dependent variable. Covariates in the core model were defined a priori based on clinical relevance.

Predictors of discordant classification at one year (CKD-EPI only < 45 vs. all equations ≥ 45) were evaluated using penalized logistic regression (Firth method) to account for separation and small-sample bias. Results are reported as odds ratios with profile-likelihood confidence intervals.

All analyses were conducted as complete-case analyses without imputation of missing data. To assess the robustness of this approach, a sensitivity analysis was performed using multiple imputation by chained equations (MICE). Twenty imputed datasets were generated. Continuous variables were imputed using predictive mean matching and binary variables using logistic regression. The imputation model included all variables from the multivariable analyses as well as the outcome variable (annual eGFR slope) to improve estimation under a missing-at-random assumption. Estimates from imputed datasets were combined using Rubin’s rules.

Statistical significance was defined as a two-sided *p*-value < 0.05. All analyses were performed using R (Version 2024.12.0 + 467).

## Results

### Study population

A total of 131 living kidney donors (55.7% female) aged ≥ 60 years at the time of donation were included in the present analyses. The median age was 64 years (IQR 62–67). Donors aged 60–64 years accounted for 55.0% (*n* = 72), those aged 65–69 years for 29.8% (*n* = 39), and donors aged ≥ 70 years for 15.3% (*n* = 20). Median follow-up after donation was 12 years (IQR 9–15). Donation was most frequently performed for offspring, with 28% of donors donating to a son and 20% to a daughter, followed by spouses (18% to wives and 17% to husbands). Baseline characteristics are summarised in Table 1.

**Table 1:** Baseline characteristics of donors aged ≥ 60 years

Characteristic	Overall	Missing
Demographics / Donor information		
Age, years	64.8 ± 3.9	
Male sex	58 (44%)	
Body mass index, kg/m²	26.4 ± 3.5	3
Donated to		
Son	37 (28%)	
Daughter	26 (20%)	
Wife	23 (18%)	
Husband	22 (17%)	
Brother	7 (5%)	
Sister	3 (2%)	
Male recipient	71 (54%)	
Donated side		4
left	65 (51%)	
right	62 (49%)	
Lifestyle		
Nicotine use		1
No	95 (73%)	
Former	20 (15%)	
Current	15 (12%)	
Active nicotine use	15 (12%)	1
Blood pressure / Hypertension		
Hypertension	85 (65%)	
Systolic blood pressure, mmHg	134.5 ± 15.9	1
Diastolic blood pressure, mmHg	82.4 ± 9.0	1
Mean arterial pressure, mmHg	99.7 ± 10.3	1
Antihypertensive medication	61 (47%)	
Number of antihypertensives		
0	70 (53%)	
1	36 (27%)	
2	17 (13%)	
3	4 (3%)	
4	4 (3%)	
Diabetes / Glycemia		
Diabetes mellitus	**0 (0%) **	
Abnormal OGTT	10 (8%)	4
Fasting glucose, mg/dL	95.0 [85.5–102.0]	
HbA1c, %	5.6 [5.3–5.9]	17
Lipids		
Hyperlipidemia	22 (17%)	
Lipid-lowering medication	17 (13%)	
Total cholesterol, mg/dL	212.0 [183.0–241.8]	9
Triglycerides, mg/dL	99.0 [72.5–143.2]	9
Kidney function / Laboratory / Urine		
Serum creatinine, mg/dL	0.8 [0.7–0.9]	
eGFR (MDRD), mL/min/1.73 m²	94.9 [83.5–105.9]	
eGFR (CKD-EPI), mL/min/1.73 m²	90.6 [80.3–95.2]	
eGFR (Cockcroft–Gault), mL/min	91.0 [78.4–105.5]	3
Creatinine clearance, mL/min	119.4 ± 32.6	
Urine albumin, mg/L	2.4 [2.4–2.4]	2
Hematuria	10 (8%)	1
Imaging / Function / Biopsy		
Kidney length (right), mm	110.0 [104.0–118.0]	6
Kidney length (left), mm	110.0 [106.0–117.0]	6
Scintigraphy MAG3 clearance (total)	214.0 [188.5–238.0]	20
Split renal function (right), %	50.0 [48.0–53.0]	11
Split renal function (left), %	50.0 [47.0–52.0]	11
Function of donated kidney	0.5 [0.5–0.5]	14
Kidney biopsy	6 (5%)	
Other Comorbidities		
Cardiovascular diseases (type 2)	21 (16%)	
Pulmonary disease	4 (3%)	
Urologic disease	10 (8%)	
Gynecologic disease	2 (2%)	
Malignancy	3 (2%)	
Mental health impairment	5 (4%)	
Thyroid disease	31 (24%)	
Thyroid medication	23 (18%)	

Continuous variables are presented as mean ± SD for selected variables and as median [IQR] otherwise; categorical variables as *n* (%).

*BMI* body mass index, *CKD-EPI* Chronic Kidney Disease Epidemiology Collaboration, *eGFR*estimated glomerular filtration rate, *HbA1c*glycated haemoglobin, *IQR*interquartile range, *MAG3*mercaptoacetyltriglycine, *MDRD* Modification of Diet in Renal Disease, *OGTT* oral glucose tolerance test, *SD* standard deviation

### Perioperative complications

**Fig. 1:**
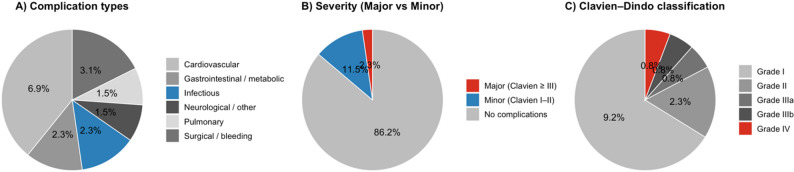
Perioperative complications in older living kidney donors. **A**) Distribution of complication types. **B**) Severity of complications classified as minor (Clavien–Dindo grades I–II) or major (Clavien–Dindo grade ≥III), with donors without complications shown separately. **C**) Distribution of complications according to Clavien–Dindo grades. Percentages refer to the proportion of donors in the total cohort. Some donors experienced more than one complication

The median postoperative hospital stay was 5 days (IQR 4–6). Postoperative complications occurred in 14.5% of donors (Fig. 1). According to the Clavien–Dindo classification, the majority of complications were minor. Major complications (Grade ≥ III) were rare and observed in 2.3% of donors.

Cardiovascular complications were the most frequent, affecting 6.9% of donors, predominantly due to postoperative hypertensive crises requiring medical management. Surgical and bleeding-related complications occurred in 3.1% of donors, including postoperative hematoma and reoperation. Infectious complications were observed in 2.3% of donors, mainly urinary tract infections and pneumonia. Pulmonary complications occurred in 1.5% of donors, including pleural effusion and atelectasis. Gastrointestinal or metabolic complications were observed in 2.3% of donors, including subileus and electrolyte disturbances. Neurological or other complications occurred in 1.5% of donors. Three donors experienced more than one complication.

### Longterm outcome in older kidney donation

Renal function assessed by CKD-EPI eGFR declined from 86.8 ± 11.3 ml/min/1.73 m² at baseline to 61.3 ± 21.0 one year after donation, 59.6 ± 3.4 ml/min/1.73 m² at 5 years and 56.8 ± 13.3 ml/min/1.73 m² at long-term follow-up (median 11.8 [IQR 8.6–14.9] years; Table 2). Overall, 37.0% of older living donors experienced an eGFR decline > 40%, whereas more pronounced declines were less frequent (> 45% in 18.5% and > 50% in 13.4%). Only two donors (1.5%) revealed an eGFR < 30 ml/in in the long-term and none of the donors required renal replacement therapy. Albuminuria at long-term follow-up was uncommon, with a median urinary albumin excretion of 4.8 mg/L (IQR 2.9–11.2) and levels ≥ 30 mg/L present in 11.0% of donors. Comorbidities at long-term follow-up included hypertension in 82.4%, hyperlipidemia in 45.8%, diabetes mellitus in 7.6%, major cardiovascular events in 6.9%, psychosomatic disorders in 4.6%, and malignancy in 19.8% (Table 3). Among these, hypertension showed the largest absolute increase over follow-up.

**Table 2:** Kidney function by estimation equation

Equation	Baseline Mean ± SD	Baseline Median (IQR)	Year 1 Mean ± SD	Year 1 Median (IQR)	LFUP Mean ± SD	LFUP Median (IQR)	Decline > 40% (Baseline→LFUP)	Decline > 45% (Baseline→LFUP)	Decline > 50% (Baseline→LFUP)
CKD-EPI	86.8 ± 11.3	90.4 (79.3–94.8)	61.3 ± 21.0	60.2 (41.7–79.4)	56.8 ± 13.3	56.3 (47.2–64.5)	37.0%	18.5%	13.4%
FAS	81.0 ± 13.8	81.3 (71.3–90.0)	54.0 ± 9.6	53.3 (46.5–59.3)	50.3 ± 11.6	50.3 (43.1–55.7)	44.0%	24.8%	14.4%
BIS1	80.1 ± 12.3	80.0 (71.8–88.0)	56.1 ± 9.0	54.6 (49.1–61.4)	51.0 ± 10.5	51.1 (45.2–56.2)	36.0%	20.0%	11.2%

Kidney function was assessed at baseline (pre-donation), one year after donation, and at long-term follow-up (LFUP) using the CKD-EPI, FAS, and BIS1 equations. Values are presented as mean ± standard deviation or median with interquartile range (IQR), as appropriate. Relative eGFR decline refers to the percentage change from baseline to long-term follow-up. Decline thresholds (> 40%, > 45%, and > 50%) were calculated separately for each estimation equation

*Abbreviations*: *eGFR* estimated glomerular filtration rate, *CKD-EPI* Chronic Kidney Disease Epidemiology Collaboration equation, *FAS* Full Age Spectrum equation, *BIS1* Berlin Initiative Study equation (version 1), *LFUP* long-term follow-up, *SD* standard deviation, *IQR* interquartile range

**Table 3:** Clinical outcomes of older living kidney donors

Metric	Donors
Follow-up, years (median [IQR])	11.8 (8.6–14.9)
Follow-up, years (range)	5.0–24.6
eGFR CKD-EPI baseline (mean ± SD)	86.8 ± 11.3
eGFR CKD-EPI 1 year (mean ± SD)	61.3 ± 21.0
eGFR CKD-EPI long-term (mean ± SD)	56.8 ± 13.3
Long-term eGFR < 45 mL/min/1.73 m²	21.8%
Long-term eGFR < 30 mL/min/1.73 m²	1.7%
Albuminuria ≥ 30 mg/L	11.0%
Relative eGFR loss > 40%	37.0%
Relative eGFR loss > 45%	18.5%
Relative eGFR loss > 50%	13.4%
Hypertension	82.4%
Hyperlipidemia	45.8%
Diabetes mellitus	7.6%
Major CV events	6.9%
Psychosomatic disorders	4.6%
Malignancy	19.8%
Death during follow-up	7.6%

Values are presented as mean ± standard deviation (SD), median with interquartile range (IQR), or percentages, as appropriate. eGFR indicates estimated glomerular filtration rate calculated using the Chronic Kidney Disease Epidemiology Collaboration (CKD-EPI) equation. Relative eGFR loss was calculated in reference to baseline eGFR. Major cardiovascular (CV) events included myocardial infarction, stroke, or coronary revascularization. Long-term eGFR refers to the last available measurement during follow-up

During follow-up, 10 donors (7.6%) died between 6 and 23 years after donation. Malignancy represented the most frequently documented cause of death (50.0% of deaths), followed by cardiovascular events (10%).

Recipient characteristics and outcomes are presented in Table 4. Recipients had a mean age of 49 ± 15 years at time of transplantation. No cases of delayed graft function were observed. During follow-up, graft failure occurred in 9.2% (12 recipients) at a median of 48 months (IQR 9–108) after transplantation, and 10.0% (13 recipients) died at a median of 44 months (IQR 9–72) after donation. Five- and ten-year death-censored graft survival was 94.6% (95% CI 90.8–98.6) and 92.9% (88.6–97.5), while overall patient survival at five and ten years was 94.5% (95% CI 90.7–98.6) and 93.7% (89.6–98.0) (Table 4).

**Table 4:** Clinical outcomes of recipients

Metric	Recipients
Delayed graft function	0%
Graft failure	9.2%
Recipient death	10.0%
5-year death-censored graft survival	94.6% (90.8–98.6)
10-year death-censored graft survival	92.9% (88.6–97.5)
5-year patient survival (95%-CI)	94.5% (90.7–98.6)
10-year patient survival (95%-CI)	93.7% (89.6–98.0)

Delayed graft function (DGF) was defined as the need for dialysis within the first 7 days post-transplantation, excluding dialysis performed solely for hyperkalaemia. Graft survival was death-censored. Patient and graft survival were estimated using Kaplan–Meier methods. CI indicates confidence interval

**Table 5:** Long-term eGFR trajectories by discordance group

Discordance group (Year 1)	*n*	Annual eGFR change after year 1 (mL/min/1.73 m²/year), median (IQR)
CKD-EPI only < 45	27	1.45 (0.58–2.24)
< 45 by all equations	6	0.85 (0.77–1.50)
Mixed classification	14	−0.88 (− 1.75–0.35)
≥ 45 by all equations	61	−1.35 (− 2.61–−0.43)

Donors were stratified according to concordance or discordance in eGFR classification below 45 mL/min/1.73 m² at one year after donation using CKD-EPI, BIS1, and FAS equations. Annual eGFR change was calculated between one year after donation and long-term follow-up and is presented as median with interquartile range (IQR). Group differences were assessed using the Kruskal–Wallis tes

*Abbreviations*: *eGFR* estimated glomerular filtration rate, *CKD-EPI* Chronic Kidney Disease Epidemiology Collaboration equation, *BIS1* Berlin Initiative Study equation (version 1), *FAS* Full Age Spectrum equation, *IQR* interquartile range, *LFUP* long-term follow-up

Overall comparison: Kruskal–Wallis test,* p *= 8.3 × 10⁻¹⁰

### Renal function trajectories and long-term decline

**Fig. 2:**
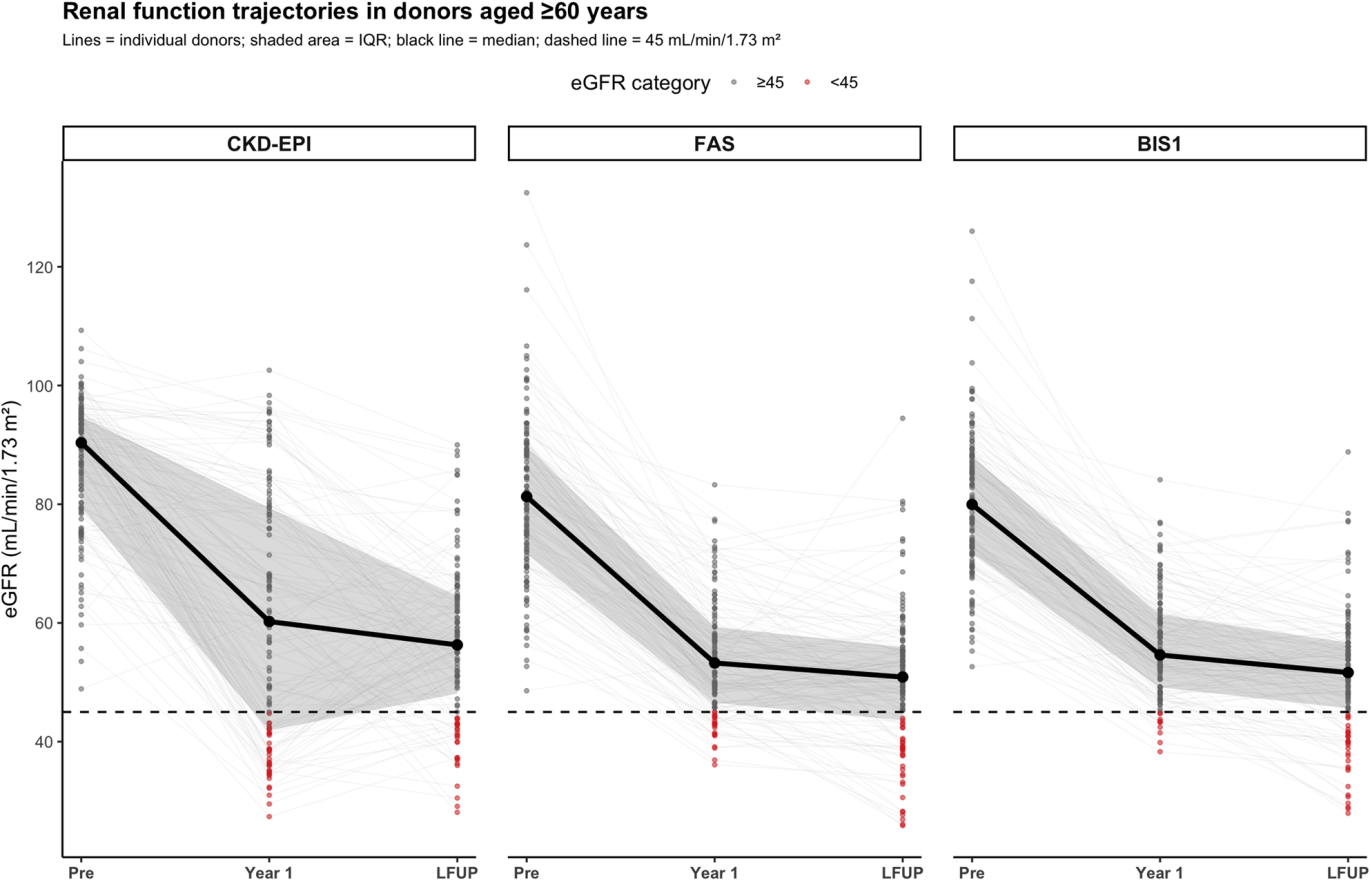
Renal function trajectories in living kidney donors aged ≥ 60 years according to eGFR estimation equation. Individual donor trajectories are shown as grey lines. The thick black line represents the median eGFR at each time point, and the shaded ribbon indicates the interquartile range (IQR). The dashed horizontal line marks the clinical threshold of 45 mL/min/1.73 m². Red points highlight individual eGFR values below this threshold (< 45 mL/min/1.73 m²). Trajectories are shown based on observed values at each time point without smoothing. Panels show results separately for BIS1, CKD-EPI, and FAS equations at pre-donation, one year after donation, and long-term follow-up.

Across all estimation equations, renal function declined markedly within the first year after donation and subsequently exhibited a flatter trajectory during long-term follow-up (Fig. 2). This biphasic pattern was observed consistently across CKD-EPI, BIS1, and FAS equations and across all age subgroups. Median eGFR values at baseline, year 1, and last follow-up are presented in Table 2.

**Fig. 3:**
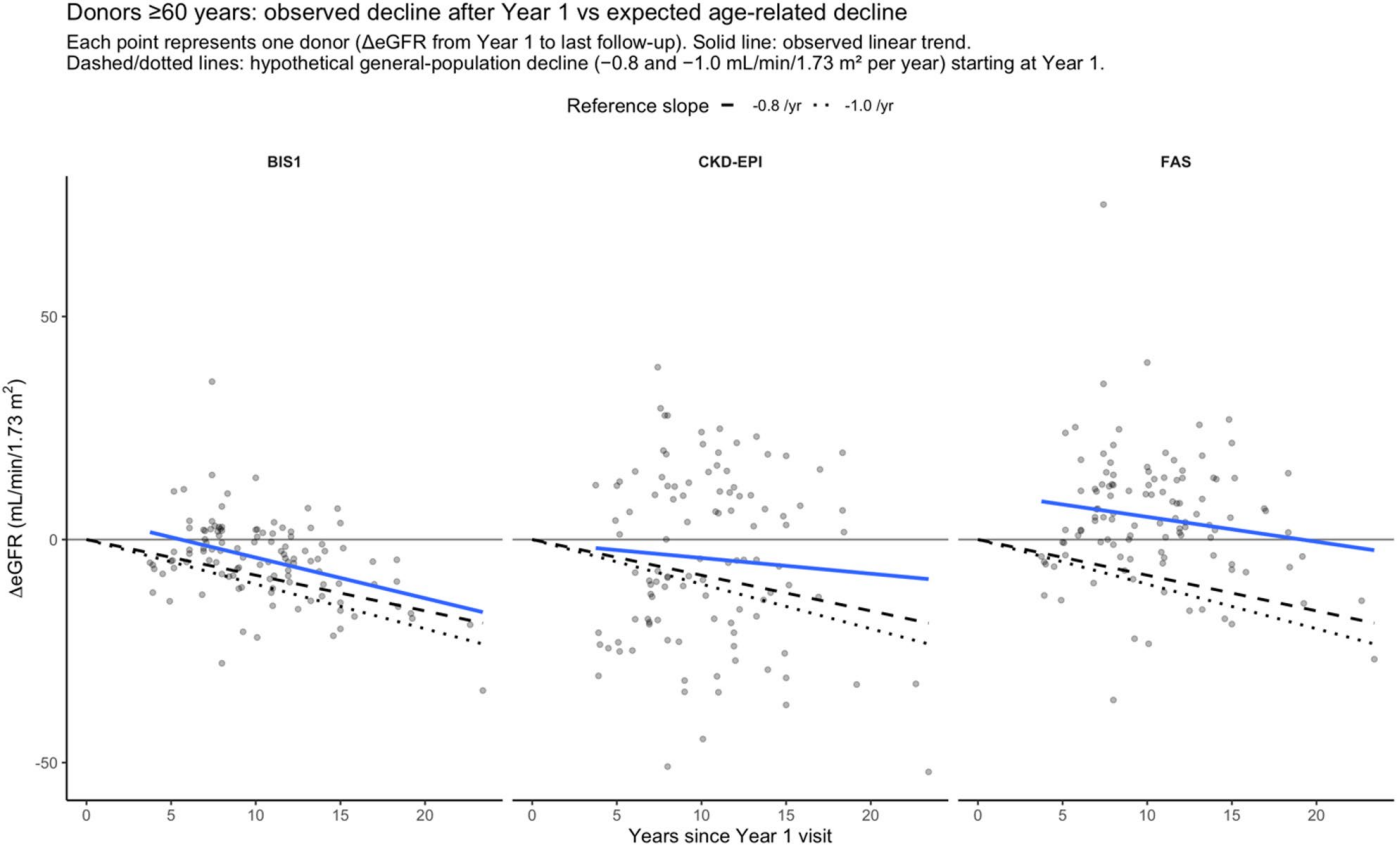
Long-term eGFR trajectory in older kidney donors compared with expected age-related decline

When analysing the period from year 1 to last follow-up, the estimated annual change in eGFR differed between equations. The estimated annual eGFR decline was modest across all equations, with the steepest decline observed for BIS1 (− 0.9 mL/min/1.73 m²/year), followed by FAS (− 0.6) and CKD-EPI (− 0.3). Individual trajectories and cohort-level trends are depicted in Fig. 3.

### Equation-dependent differences in eGFR levels and classification

**Fig. 4:**
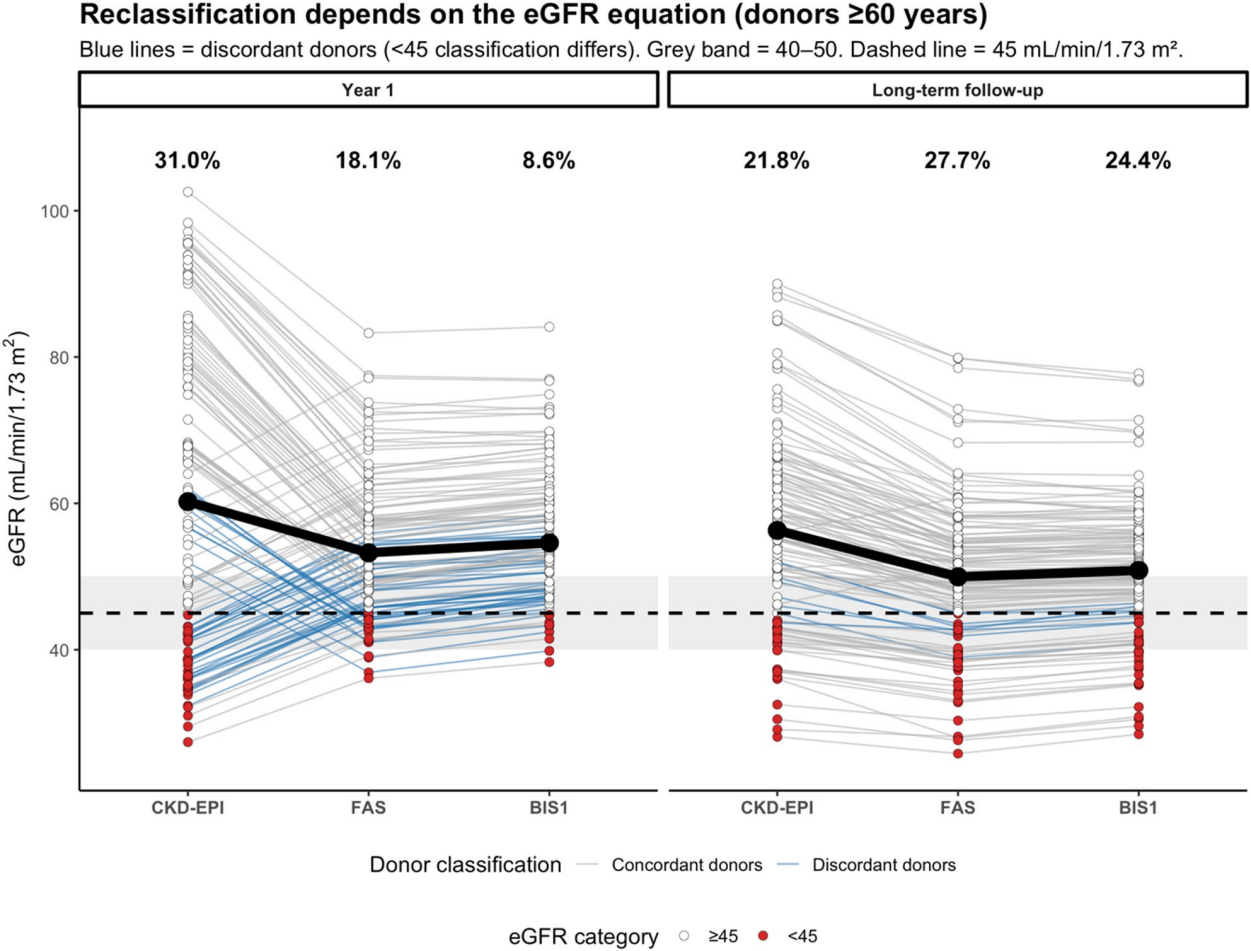
Reclassification of kidney function depends on the eGFR equation in donors aged ≥ 60 years. Individual eGFR values one year after donation and at long-term follow-up are shown for CKD-EPI, FAS, and BIS1 equations. Each line represents an individual donor. Grey lines indicate concordant classification across equations, and blue lines indicate discordant classification with respect to the threshold of 45 mL/min/1.73 m². Red dots indicate eGFR < 45 mL/min/1.73 m², and open circles indicate eGFR ≥ 45 mL/min/1.73 m². The dashed line denotes the threshold of 45 mL/min/1.73 m², and the shaded band indicates the range between 40 and 50 mL/min/1.73 m². Bold black lines represent cohort-level median eGFR values. Percentages above panels indicate the proportion of donors classified as eGFR < 45 mL/min/1.73 m² by each equation.

Estimated glomerular filtration rate (eGFR) differed across estimation equations at all time points (Table 2). At baseline, mean eGFR was highest with CKD-EPI and lower with FAS and BIS1. These equation-dependent differences resulted in substantial variation in clinical classification below the threshold of 45 mL/min/1.73 m², most pronounced one year after donation (Fig. 4). At this time point, 31.0% of donors were classified below the threshold using CKD-EPI, compared with 18.1% using FAS and 8.6% using BIS1. At long-term follow-up, classification rates converged but remained discordant across equations.

**Fig. 5:**
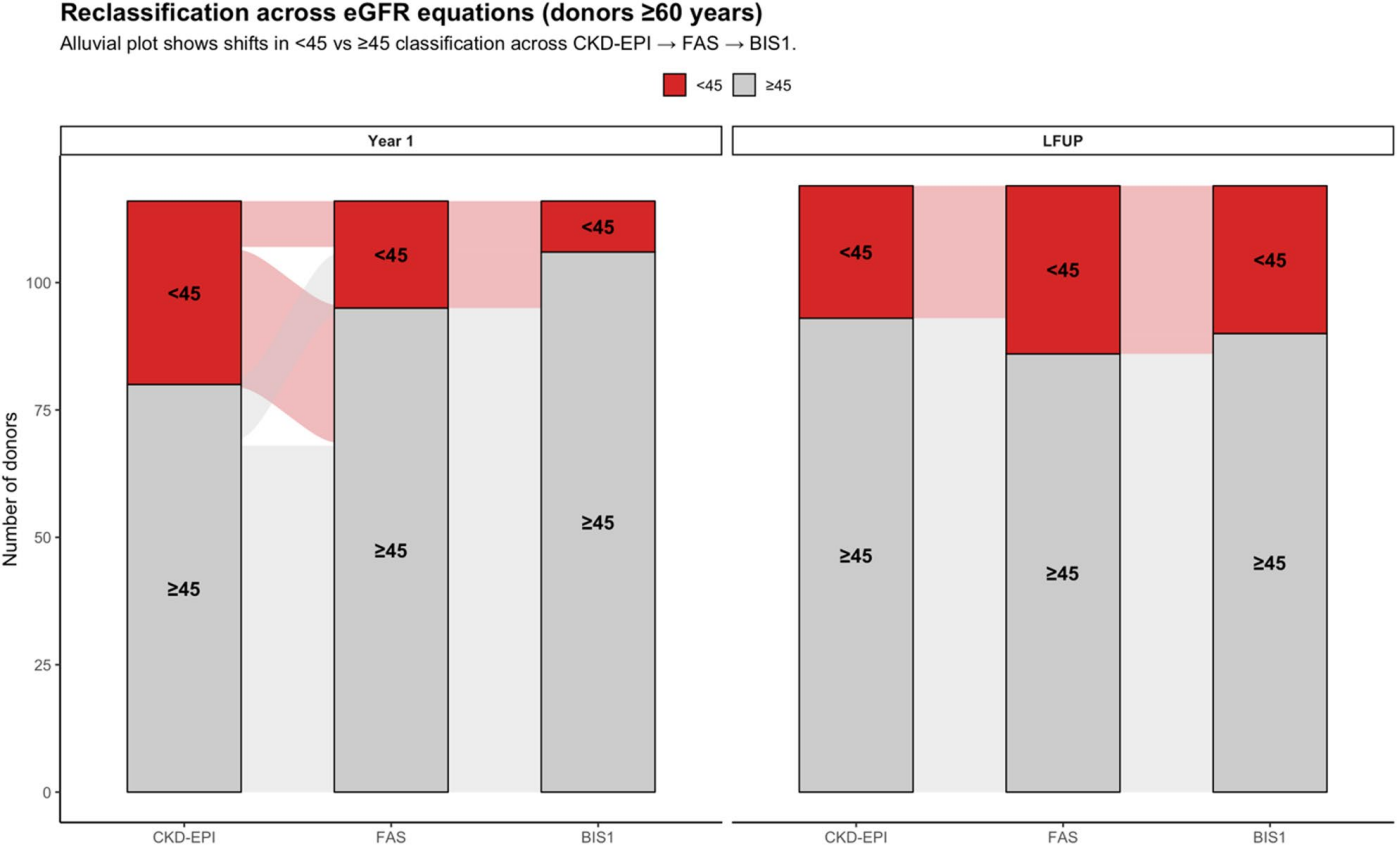
Reclassification across eGFR equations in donors aged ≥ 60 years. Alluvial plot illustrating shifts in classification (< 45 vs. ≥ 45 mL/min/1.73 m²) across CKD-EPI, FAS, and BIS1 equations at one year after donation and at long-term follow-up. Bars represent the number of donors classified in each eGFR category, and flows indicate transitions between categories across equations. Grey indicates eGFR ≥ 45 mL/min/1.73 m², and red indicates eGFR < 45 mL/min/1.73 m².

Concordance between equations was lowest one year after donation, with 23.3% of donors classified below the threshold exclusively by CKD-EPI and 12.9% showing discordant classification. At long-term follow-up, concordant classification increased and discordance decreased to 6.7% (Fig. 5). Notably, donors classified below 45 mL/min/1.73 m² exclusively by CKD-EPI showed stable long-term renal trajectories despite being classified below the threshold (Table 5).

To quantify equation-dependent differences beyond threshold-based classification, we additionally assessed relative eGFR loss from baseline to long-term follow-up. The proportion of donors with an eGFR decline > 40% differed across equations, occurring in 37.0% with CKD-EPI, 44.0% with FAS, and 36.0% with BIS1 (Table 2).

### Renal adaptation at year 1 and long-term trajectories

Renal adaptation at 1 year after donation was defined as the ratio of eGFR at year 1 to baseline and categorized as good (≥ 70%), intermediate (60–69%), or poor (< 60%). Donors with poorer adaptation showed a significantly faster decline in eGFR from year 1 to last follow-up (*p* < 0.001). Lower adaptation was also associated with a higher proportion of substantial long-term eGFR loss (> 40%), although this trend did not reach statistical significance (*p* = 0.085).

### Albuminuria in donors with preserved long-term eGFR

Among donors with preserved long-term kidney function (eGFR ≥ 45 mL/min/1.73 m²), elevated urinary albumin excretion at long-term follow-up (≥ 30 mg/L) was associated with a significantly steeper annual eGFR decline compared with donors without albuminuria (median − 2.10 vs. +0.22 mL/min/1.73 m²/year; *p* = 0.015). Despite similar eGFR categories, donors with albuminuria showed a numerically higher prevalence of substantial long-term eGFR loss (> 40%) (37.5% vs. 21.2%; *p* = 0.37).

### Characteristics of donors with rapid decline of renal function

Accelerated decline in kidney function was assessed using annual changes in eGFR between one year after donation and long-term follow-up. Using a threshold of > 3 mL/min/1.73 m²/year, the prevalence of rapid decline differed markedly across estimation equations. Based on CKD-EPI, 10.2% of donors met the criterion for accelerated decline, whereas only 1.0% were classified as rapid decliners using FAS and 1.0% using BIS1. Concordant classification across all equations was observed in 88.9% of donors (non-rapid by all equations) and in 1.9% (rapid by all equations). In contrast, 9.3% of donors were classified as rapid decliners exclusively by CKD-EPI, while no donors were classified exclusively by FAS or BIS1.

Compared with donors with stable trajectories, rapid progressors had a higher body mass index (27.8 vs. 26.2 kg/m²) and a higher prevalence of baseline hypertension (72.7% vs. 64.9%).

Older donors with a long-term eGFR loss > 40% after donation more frequently had pre-existing hypertension at baseline (77.3% vs. 56.0%, *p* = 0.033), whereas age, BMI, baseline renal function, metabolic parameters, and other cardiovascular risk factors were comparable between groups. At long-term follow-up, hypertension remained more prevalent among donors with a long-term eGFR loss > 40% (93.2% vs. 76.0%, *p* = 0.024), while the incidence of cardiovascular events, diabetes mellitus, hyperlipidemia, and malignancy did not differ.

### Determinants of long-term eGFR decline

Multivariable linear regression analyses were performed to identify factors associated with annual eGFR decline after year 1. In the core model, female sex (β = −1.88, 95% CI − 2.90 to − 0.86), higher body mass index (β = −0.084 per kg/m², 95% CI − 0.17 to − 0.00), baseline hypertension (β = −0.60, 95% CI − 1.19 to − 0.01), and lower eGFR at year 1 (β = −0.048 per mL/min/1.73 m², 95% CI − 0.073 to − 0.024) were associated with steeper long-term decline. Age at donation was not significantly associated with decline. Extended models including cardiovascular and metabolic variables did not identify additional factors associated with long-term decline. Full regression results are shown in Supplementary Table S1.

## Discussion

In this cohort of living kidney donors aged ≥ 60 years with extended long-term follow-up, several clinically relevant insights emerge regarding clinical outcome, risk stratification, post-donation renal trajectories, and the interpretation of kidney function in older donors. While previous studies have established the safety of living kidney donation in older individuals, they did not address how post-donation kidney function should be interpreted in clinical practice [6, 29–32]. In our cohort, the proportion of donors classified below the commonly used threshold of 45 mL/min/1.73 m² varied substantially depending on the eGFR Eq. (31.0% vs. 18.1% vs. 8.6% at one year), despite similar longitudinal trajectories. Importantly, donors classified below this threshold by a single equation exhibited favourable long-term renal function, indicating that threshold-based classification does not necessarily reflect clinically meaningful risk.

More than one third of older donors experienced a long-term eGFR decline > 40%, whereas only a small proportion showed a decline > 50%. Importantly, the proportion of donors classified as having clinically relevant impairment varied markedly depending on the estimation equation applied. Across all estimation equations, renal function followed a characteristic biphasic pattern, with an early decline within the first year after donation followed by long-term stabilisation, as described previously in living kidney donors [33, 34]. Progressive kidney disease was uncommon, even at advanced age. The observed annual eGFR decline after year 1 was modest and in part comparable to, or even lower than, rates reported in age-matched individuals from the general population [20, 35]. This finding suggests that nephrectomy does not necessarily accelerate loss of kidney function in older donors and is most plausibly explained by a healthy donor effect, whereby living kidney donors represent a highly selected population with favourable baseline health [36–38].

Chronological age itself did not emerge as a determinant of adverse long-term renal outcome. Donors aged ≥ 70 years did not exhibit steeper annual eGFR decline, higher rates of substantial long-term eGFR loss, or worse clinical outcomes compared with younger older donors in line with previous observational studies [6, 39–41]. These findings support the concept that advanced age per se should not be considered a contraindication to living kidney donation in appropriately selected individuals.

Beyond biological effects, our data demonstrate that clinical interpretation of post-donation kidney function is strongly influenced by the estimation equation applied. Discrepancies among creatinine-based equations assessing glomerular filtration rate have been detected in older adults [42, 43]. It should be noted that the CKD-EPI eGFR—the formula most frequently used in clinical practice for donor assessment—was the basis for the present study, whereas FAS and BIS1 were applied retrospectively for the purposes of this investigation. Consequently, donor selection relied primarily on renal function estimates derived using CKD-EPI; this likely explains, at least in part, the higher baseline eGFR values determined by CKD-EPI compared to age-adapted formulas and should be taken into account when interpreting these differences. Beyond these differences in baseline values, significant discrepancies in post-donation classification was also observed, depending on the specific formula used. One year after donation, CKD-EPI classified substantially more donors below the clinically relevant threshold of 45 mL/min/1.73 m² than age-adapted equations, a phenomenon reported previously in older populations [44].

Importantly, in the absence of measured GFR, it is not possible to determine which equation most accurately reflects true kidney function in living kidney donors. All evaluated equations were developed in populations with two functioning kidneys and have not been specifically validated in the setting of kidney donation. Therefore, observed differences should be interpreted as equation-dependent variation in classification rather than true differences in renal function or risk. In this context, commonly used thresholds such as 45 mL/min/1.73 m² cannot be assumed to carry equivalent clinical meaning across equations. As no validated eGFR thresholds exist for living kidney donors, this cut-off should be interpreted as a pragmatic reference rather than a definitive indicator of risk. Consistent with this, donors classified below this threshold exclusively by CKD-EPI exhibited favourable long-term trajectories, suggesting that such classifications often do not reflect clinically meaningful disease progression. In contrast, donors consistently classified below the threshold across all equations showed steeper long-term decline, indicating that concordant classification identifies a subgroup with genuinely increased renal vulnerability [44]. These findings highlight the limitations of single time-point eGFR thresholds in older donors and emphasise the need for longitudinal assessment rather than cross-sectional categorisation. From a clinical perspective, this implies that donor follow-up and risk stratification should not rely solely on single eGFR threshold values, but should incorporate longitudinal trajectories, early post-donation adaptation, and additional markers such as albuminuria. Therefore, classifying potential donors according to fixed eGFR thresholds has to be questioned [45]. Other guidelines favour age-dependent decision [46]. The present results demonstrate that analyses focusing on renal trajectories provided more clinically meaningful information for risk stratification. Early renal adaptation, assessed by the relative recovery of eGFR one year after donation, emerged as a key integrative marker of functional reserve. Donors with limited adaptation at year 1 exhibited significantly steeper long-term eGFR decline and were more likely to experience substantial loss of renal function, independent of age. This suggests that early post-donation renal adaptation captures interindividual differences in compensatory capacity and may precede later decline. From a clinical point of view, eGFR at one year after donation represents a pragmatic and clinically relevant time point to identify donors who may benefit from closer long-term monitoring. Albuminuria further refined risk stratification among donors with preserved long-term eGFR. Even in donors with eGFR ≥ 45 mL/min/1.73 m², the presence of albuminuria was associated with a significantly steeper annual decline in kidney function, despite similar eGFR categories. This observation reinforces the concept that albuminuria reflects ongoing renal stress or structural vulnerability that is not captured by eGFR alone.

In this context, hypertension emerged as the most clinically relevant long-term comorbidity. Both baseline and persistent hypertension were associated with steeper long-term eGFR decline, whereas chronological age was not. These findings suggest that blood pressure acts as a modifiable amplifier of limited renal reserve and highlight long-term blood pressure control as a key target in post-donation follow-up of older donors [47]. Together, these results underscore the importance of longitudinal risk assessment and active management of modifiable cardiometabolic risk factors rather than reliance on baseline characteristics or cross-sectional eGFR thresholds alone.

This study has several limitations. The cohort represents a highly selected population of living kidney donors, reflecting a typical “healthy donor effect”, as all donors underwent rigorous pre-donation screening and had favourable baseline health profiles. Therefore, the observed stability of renal function and low rate of clinically relevant decline should be interpreted within this context and may not be generalisable to more heterogeneous or comorbid populations. In addition, no measured GFR was available, and all equations were applied outside their original validation setting. The single-centre design and moderate sample size further limit external validity and statistical power. These analyses should therefore considered hypothesis-generating. Despite these limitations, the study provides long-term follow-up with a trajectory-based assessment of kidney function and a direct comparison of multiple eGFR equations within the same cohort, enabling a clinically relevant evaluation of equation-dependent differences in classification.

## Conclusions

In conclusion, the perioperative outcomes in this older living kidney donor cohort were favourable, with a low rate of major complications, supporting the clinical feasibility of living kidney donation in carefully selected older individuals. In older living kidney donors, kidney function is generally stable after donation, and progressive kidney disease is rare. However, the choice of eGFR estimation equation has a significant impact on the clinical classification and interpretation of kidney trajectories. The observed rate of renal function loss is modest and comparable to that in the general population, consistent with a healthy donor effect. These findings support the integration of age-adapted estimation equations and longitudinal studies into post donation follow-up and underscore the need to reconsider current eGFR-based risk stratification strategies in older donors. Future studies should focus on validating eGFR estimation using measured GFR and on developing donor-specific, trajectory-based risk models to improve long-term clinical management in this population. 

## Supplementary Information

Supplementary Material 1: Table S1. Predictors of post-donation eGFR decline in living kidney donors aged ≥60 years. Table S2. Sensitivity Analysis Using Multiple Imputation.

## Data Availability

The data that support the findings of this study are available from the HeiKiD study group but restrictions apply to the availability of these data, which were used under license for the current study, and so are not publicly available. Data are however available from the authors upon reasonable request and with permission of the HeiKiD study group.

## Abbreviations

BIS1: Berlin Initiative Study
CKD-EPI: Chronic Kidney Disease
eGFR: estimated Glomerular Filtration Rate
FAS: Full Age Spectrum
GFR: Glomerular Filtration Rate
IQR: Interquartile Range
LD: Living Donor
LKD: Living Kidney Donation
