# Supplementary material for: Long-term kidney outcomes after living donation in older adults: clinical findings and equation-dependent eGFR estimates
Source: BMC Geriatr. 2026 Jul 7;26:926. doi: 10.1186/s12877-026-07826-8 (PMC13344051; doi:10.1186/s12877-026-07826-8)
Supplement: Supplementary file 1 — Supplementary Material 1: Table S1. Predictors of post-donation eGFR decline in living kidney donors aged ≥60 years. Table S2. Sensitivity Analysis Using Multiple Imputation. [file 12877_2026_7826_MOESM1_ESM.docx]

**Supplementary Material**

| **Predictor** | **Model 1 (Core) β (95 % CI)** | **p** | **Model 2 (Extended) β (95 % CI)** | **p** |
| --- | --- | --- | --- | --- |
| Intercept | 9.92 (4.48, 15.37) | <0.001 | 9.98 (4.07, 15.89) | 0.001 |
| Age at donation (per year) | −0.06 (−0.13, 0.01) | 0.096 | −0.06 (−0.13, 0.02) | 0.126 |
| Female sex (vs male) | −1.88 (−2.90, −0.86) | <0.001 | −1.89 (−2.98, −0.80) | 0.001 |
| BMI (per kg/m²) | −0.08 (−0.17, −0.00) | 0.046 | −0.08 (−0.17, 0.00) | 0.063 |
| Hypertension at baseline (yes vs no) | −0.60 (−1.19, −0.01) | 0.047 | −0.52 (−1.21, 0.17) | 0.135 |
| eGFR at Year 1 (per mL/min/1.73 m²) | −0.05 (−0.07, −0.02) | <0.001 | −0.05 (−0.07, −0.02) | <0.001 |
| Mean arterial pressure (per mmHg) | — | — | −0.01 (−0.03, 0.02) | 0.732 |
| Smoking at baseline (yes vs no) | — | — | 0.02 (−0.35, 0.39) | 0.922 |
| Hyperlipidaemia at baseline (yes vs no) | — | — | 0.16 (−0.65, 0.96) | 0.701 |
| Coronary artery disease (yes vs no) | — | — | −0.49 (−2.31, 1.34) | 0.597 |
| Peripheral arterial disease (yes vs no) | — | — | 1.01 (−2.36, 4.37) | 0.555 |
| Annual change in estimated glomerular filtration rate (eGFR slope) after donation was calculated for each donor using linear mixed-effects modeling with random intercepts, based on all available post-donation eGFR measurements. eGFR was estimated using the CKD-EPI equation. Values are β coefficients from linear regression with 95 % confidence intervals. Model 1 adjusted for age, sex, BMI, baseline hypertension, and year-1 eGFR. Model 2 additionally adjusted for mean arterial pressure, smoking, hyperlipidaemia, coronary artery disease, and peripheral arterial disease. Negative β values indicate faster annual eGFR decline.  **Abbreviations:** eGFR, estimated glomerular filtration rate; CKD-EPI, Chronic Kidney Disease Epidemiology Collaboration; FAS, Full Age Spectrum; BMI, body mass index; MAP, mean arterial pressure; CI, confidence interval; IQR, interquartile range; DM, diabetes mellitus; PAVK, peripheral arterial disease. | | | | |

**Supplementary Table S1.**

Predictors of post-donation eGFR decline in living kidney donors aged ≥60 years

**Supplementary Table S2**

**Sensitivity analysis using multiple imputation.**

Comparison of regression results between complete-case analysis and multiple imputation for predictors of post-donation eGFR decline in living kidney donors aged ≥60 years.

| **Predictor** | **Complete-case β (95% CI)** | **p-value** | **Multiple imputation β (95% CI)** | **p-value** |
| --- | --- | --- | --- | --- |
| Intercept | 9.87 (4.40, 15.34) | <0.001 | 9.88 (4.46, 15.29) | <0.001 |
| Age at donation (per year) | −0.06 (−0.13, 0.01) | 0.102 | −0.06 (−0.13, 0.01) | 0.094 |
| Female sex (vs male) | −1.82 (−2.85, −0.79) | <0.001 | −1.89 (−2.91, −0.88) | <0.001 |
| BMI (per kg/m²) | −0.09 (−0.17, −0.00) | 0.043 | −0.08 (−0.17, −0.00) | 0.045 |
| Hypertension at baseline (yes vs no) | −0.63 (−1.23, −0.04) | 0.038 | −0.60 (−1.18, −0.02) | 0.044 |
| eGFR at Year 1 (per mL/min/1.73 m²) | −0.05 (−0.07, −0.02) | <0.001 | −0.05 (−0.07, −0.02) | <0.001 |
| Baseline albuminuria (per mg/L) | 0.01 (−0.09, 0.10) | 0.900 | 0.01 (−0.09, 0.10) | 0.888 |
| Legend: Annual change in estimated glomerular filtration rate (eGFR slope) after donation was calculated as the difference between eGFR at one year and long-term follow-up, standardised per year. eGFR was estimated using the CKD-EPI equation. Values are β coefficients from multivariable linear regression models with 95% confidence intervals (CI). The same model specification was applied in both analyses, including age at donation, sex, body mass index (BMI), baseline hypertension, eGFR at one year, and baseline albuminuria.  The complete-case analysis included only donors with complete data for all covariates. Multiple imputation was performed using chained equations (m=20). Continuous variables were imputed using predictive mean matching and binary variables using logistic regression. The imputation model included all covariates and the outcome variable (eGFR slope). Estimates from imputed datasets were pooled using Rubin’s rules.  The consistency between complete-case and multiple imputation results indicates robustness of the findings to missing data.  Abbreviations: eGFR, estimated glomerular filtration rate; CKD-EPI, Chronic Kidney Disease Epidemiology Collaboration; BMI, body mass index; CI, confidence interval. | | | | |
